# HIF-1α Promotes Macrophage Extracellular Trap Formation and Exacerbates Acute Lung Injury in Neonatal Sepsis

**DOI:** 10.3390/biomedicines14051145

**Published:** 2026-05-18

**Authors:** Huiling Zhang, Wei Huang, Xinlong Dai, Jundi Zheng, Xinyao Jiang, Yutao Yang, Hanhui Zhong, Guang Yang

**Affiliations:** 1 Senior Department of Pediatrics, Chinese PLA General Hospital, Beijing 100700, China; alin2126@163.com; 2 Department of Critical Care Medicine, Southern Medical University, Guangzhou 510080, China; wei11920012@163.com; 3 The Department of Anesthesiology, Guangdong Medical University, Zhanjiang 524001, China; 17398201523@163.com (X.D.); m13990081030_1@163.com (X.J.); 18027752068@163.com (Y.Y.); 4 The Department of Respiratory Medicine, Guangdong Provincial of Integrated Chinese and Western Medicine, Foshan 528200, China; 18218522163@163.com

**Keywords:** neonatal sepsis, acute lung injury, macrophage extracellular trap, HIF-1α, ENO2, glycolysis

## Abstract

**Background**: Acute lung injury (ALI) is a major contributor to mortality in neonatal sepsis, yet the mechanisms underlying early lung damage remain incompletely understood. Although extracellular traps (ETs) have been implicated in inflammatory injury, the cellular origin and regulatory pathways of ET formation in neonatal sepsis remain unclear. This study aimed to determine the source of ETs and to investigate the role of hypoxia-inducible factor-1α (HIF-1α) in regulating macrophage extracellular traps (METs) formation and lung injury. **Methods**: Neonatal sepsis was induced in mice by intraperitoneal injection of cecal slurry. METs formation was assessed by immunofluorescence staining, Western blotting, and extracellular DNA quantification. Selective depletion of macrophages or neutrophils was performed to determine the cellular source of ETs. In vitro experiments were conducted using macrophages stimulated with lipopolysaccharide or phorbol 12-myristate 13-acetate. RNA sequencing analysis and pharmacological inhibition were used to examine the roles of HIF-1α, glycolysis, and enolase 2 (ENO2) in METs formation, lung injury, and survival outcomes. **Results**: We identify macrophages as a predominant source of ETs in the lung and demonstrate that METs contribute to lung injury in neonatal sepsis. Depletion of macrophages or pharmacological inhibition of METs formation markedly attenuated lung injury and improved survival in neonatal sepsis mice. Mechanistically, we suggest that HIF-1α promotes METs formation by driving glycolysis in macrophages. Furthermore, this process appears to involve the upregulation of key glycolytic enzymes, including ENO2, potentially facilitating METs release. In turn, METs are implicated in enhancing macrophage inflammatory activation, which could exacerbate lung injury. Importantly, pharmacological targeting of HIF-1α pathways reduces METs formation, attenuates lung inflammation, and improves survival outcomes. **Conclusions**: These findings suggest a role for HIF-1α in regulating METs formation and support that targeting this pathway could represent a potential therapeutic strategy for neonatal sepsis-associated acute lung injury.

## 1. Introduction

Neonatal sepsis is a leading cause of morbidity and mortality in the first month of life, with an estimated annual incidence of millions of cases worldwide and a substantial contribution to global childhood mortality [1,2]. Among its complications, acute lung injury (ALI) commonly occurs and is closely associated with poor clinical outcomes, contributing substantially to sepsis-related mortality in neonates [3,4]. Despite advances in supportive care, the molecular mechanisms driving sepsis-associated lung injury in neonates remain poorly defined, limiting the development of effective targeted therapies.

The dysregulated release of extracellular traps (ETs) represents an important effector mechanism of the innate immune system with dual roles in host defense and pathology, including lung injury. ETs are structures composed of decondensed chromatin (DNA and histones) and decorated with antimicrobial granular proteins, which are expelled by activated leukocytes to immobilize pathogens [5]. Initially characterized in neutrophils as neutrophil extracellular traps (NETs), this process is now recognized to occur in other immune cells, including monocytes and macrophages, which release macrophage extracellular traps (METs) [5,6,7]. Although NETs were the first to be described and most extensively studied, growing research attention is now focused on METs. Excessive or misplaced formation of METs can drive tissue injury and inflammation in various diseases, such as cancer metastasis, acute kidney injury, and asthma, largely through the cytotoxic effects of components like extracellular histones [8,9,10,11]. However, whether METs participate in organ-specific inflammatory responses during neonatal sepsis remains largely unknown.

In the specific context of neonatal sepsis-induced lung injury, macrophages have been identified as central mediators of the inflammatory cascade [12,13,14,15]. Their pivotal role is further underscored by therapeutic evidence, for instance, the cold-inducible RNA-binding protein (CIRP)-derived peptide C23, which inhibits macrophage TNF-α production, has been shown to ameliorate lung inflammation in this setting [16]. Given that macrophages are capable of releasing METs, an immune effector mechanism, it is plausible that METs constitute a previously overlooked pathway through which macrophages exacerbate lung injury. However, the specific contribution of METs to the pathogenesis of neonatal sepsis-associated acute lung injury remains largely undefined.

In this study, we investigated whether macrophage-derived ETs contribute to neonatal sepsis-induced ALI and explored the metabolic mechanisms regulating their formation. We demonstrate that METs play an important role in lung injury following neonatal sepsis and identify a hypoxia-inducible factor-1α (HIF-1α)–enolase 2 (ENO2)–dependent glycolysis as a key regulatory axis regulating METs formation. Pharmacological inhibition of HIF-1α–ENO2–glycolysis–mediated METs formation may thus represent a viable therapeutic strategy for neonatal sepsis-associated lung injury.

## 2. Materials and Methods

### 2.1. Mice

The animal protocols in this study were reviewed and approved by the Animal Care and Use Committee of the Affiliated Hospital of Guangdong Medical University, following the NIH Guide for the Care and Use of Laboratory Animals (approval number AHGDMU-LAC-B-202509-0088). Neonatal C57BL/6J mice, aged between 5 and 7 days (postnatal), were sourced from the Animal Center of Guangdong Medical University (Zhanjiang, China). Throughout the study, the mice were housed in a specific pathogen-free (SPF) environment with a 12 h light/dark cycle and provided with ad libitum access to water and a standard diet. During the experimental procedures, neonatal mice were separated from their mothers in groups and placed on a warming blanket. After the procedure, littermates were returned to their mother until the experiment’s completion. Mice of both sexes were used in this study.

### 2.2. Neonatal Sepsis Model

Neonatal sepsis model was induced by intraperitoneal administration of cecal slurry (CS), following previously reported protocols [17]. Cecal slurry donors were obtained from adult male C57BL/6 mice (6–8 weeks old). Following euthanasia, cecal contents were collected aseptically, weighed, and suspended in sterile 5% dextrose solution to 80 mg/mL, subsequently filtered using a 70-μm mesh and thoroughly mixed to ensure a homogeneous preparation. Neonatal recipient mice received intraperitoneal injections of CS at 10 µL/g body weight. Mice were observed every 6 h, and survival was recorded. To determine whether mortality required live bacteria, CS was heat-inactivated (72 °C for 10 min) in parallel experiments [18]. Mice were subjected to the following treatments: mice were injected with 5 mg/kg DNase I (10104159001, Roche, Basel, Switzerland) just before injection of cecal slurry solutions, 10 mg/kg/day chloride amidine (CLA, pan-PAD inhibitor to block ET formation, Cayman Chemical, Ann Arbor, MI, USA) subcutaneously (s.c.) or PBS as control for 3 days before cecal slurry solutions injection, 20 mg/kg LW6 (HIF-1α inhibitor, HY-13671; MedChemExpress (MCE), Monmouth Junction, NJ, USA) intraperitoneally (i.p.) 30 min before cecal slurry solutions injection, 20 mg/kg POMHEX (ENO2 inhibitor, HY-131904; MedChemExpress (MCE), Monmouth Junction, NJ, USA) i.p. for 3 days prior to cecal slurry solutions injection. Neonatal mice from different litters were randomly assigned to experimental groups using a random number-based method. Investigators responsible for outcome assessment were blinded to group allocation.

### 2.3. Depletion of Macrophages and Neutrophils

Macrophage depletion was achieved by intraperitoneal administration of 60 μL clodronate liposomes (HY-172202; MedChemExpress (MCE), Monmouth Junction, NJ, USA), with PBS liposomes used as controls, 24 h prior to CS injection. To deplete neutrophils, mice were assigned to receive an intraperitoneal injection of 50 μg of anti-Ly6G antibody (BP0075-1, Bio X Cell, Lebanon, NH, USA) 24 h before CS injection.

### 2.4. Bronchoalveolar Lavage Fluid (BALF) Protein and DNA Concentration Analysis

After anesthesia, the trachea was carefully exposed and intubated, followed by bronchoalveolar lavage using 600 μL sterile PBS, which was gently instilled and aspirated for three consecutive cycles. The recovered lavage fluid was then centrifuged (300× *g*, 10 min) to separate cellular components, and the supernatant fraction was collected for subsequent analyses. Total protein content in BALF was determined using a bicinchoninic acid (BCA) assay kit (P0012, Beyotime Biotechnology, Shanghai, China). Extracellular DNA concentration was assessed with the Quant-iT PicoGreen dsDNA assay (P11496, Invitrogen, Carlsbad, CA, USA) following standard procedures [19].

### 2.5. Lung Wet/Dry Weight Measurement

The extent of pulmonary edema was evaluated by determining the ratio of lung wet mass to dry mass [20]. Briefly, freshly isolated lung samples were weighed immediately to record the initial (wet) weight, followed by dehydration in an oven at 80 °C for 48 h until a constant dry weight was achieved. The wet/dry ratio was then calculated for each sample.

### 2.6. Enzyme-Linked Immunosorbent Assay (ELISA)

ELISA kits for TNF-α (88-7324-88, Thermofisher Scientific, Waltham, MA, USA) and Il1β (88-7013A-88, Thermofisher Scientific) were used to measure the cytokine levels in lung tissue according to the manufacturer’s instructions.

### 2.7. Hematoxylin and Eosin (H&E) Staining and Scoring

To evaluate lung architecture and injury, left lung lobes were preserved in 4% paraformaldehyde and embedded in paraffin blocks. Lung sections (3 μm thick) were prepared and subjected to hematoxylin and eosin (H&E) staining. Histopathological assessment was performed by two independent pathologists who remained blinded to the experimental groups. Lung injury was quantified using a validated semi-quantitative scoring system [21]. This assessment encompassed four key pathological features: infiltration of neutrophils into interstitial and alveolar spaces, formation of hyaline membranes, presence of proteinaceous debris within the airspaces, and the degree of alveolar septal thickening.

### 2.8. Cell Culture

Murine macrophage RAW264.7 (C7505, Beyotime Biotechnology, Shanghai, China) cells were cultured in DMEM enriched with 10% FBS and maintained at 37 °C in a 5% CO_2_ incubator.

### 2.9. Induction of RAW264.7 Cells METs and Treatment Conditions

To induce METs formation on RAW264.7 cells, 1 × 10^6^ cells/mL RAW264.7 cells were cultured in DMEM medium pretreated with 10 U/mL DNase I (10104159001, Roche Diagnostics, Basel, Switzerland), 200 μM PAD inhibitor CLA (10599-5, Cayman Chemicals, Ann Arbor, MI, USA), 50 mM 2DG (D6134, Sigma-Aldrich, Taufkirchen, Germany), 10 μM POMHEX (HY-131904, MCE), 20 µM LW6 (HY-13671, MCE) or 10 μM AP-III-a4 (HY-15858A, MCE) for 1 h, and stimulated with 100 nM PMA (HY-18739, MCE) for 6 h or 100 ng/mL LPS (L2880, Sigma-Aldrich, Taufkirchen, Germany) for 12 h. Supernatants were collected for extracellular DNA quantification, while cells were processed for analysis of MET-associated markers.

### 2.10. Western Blot Analysis

Total proteins from lung tissues and RAW264.7 cells were extracted by homogenizing samples in RIPA lysis buffer containing a cocktail of phosphatase and protease inhibitors. Following denaturation at 95 °C for 5 min, protein concentrations were normalized, and equal quantities were separated via 8–15% SDS-PAGE. The proteins were then immobilized onto PVDF membranes through electro-transfer. To prevent non-specific binding, membranes were blocked for 1 h at room temperature using 5% non-fat milk. Primary antibody incubation, including rabbit anti-Histone H3 (ab5103, Abcam, Cambridge, UK) and mouse anti-MPO (DF2651, Affinity Biosciences, Cincinnati, OH, USA), was conducted overnight at 4 °C. Subsequently, the membranes were treated with appropriate secondary antibodies (HRP-conjugated Goat anti-Mouse (AS080, ABclonal Technology, Wuhan, China) or anti-Rabbit IgG (AS014, ABclonal)) and visualized using an enhanced chemiluminescence kit on a Tanon 5200 imaging system.

### 2.11. RNA Isolation and Quantitative PCR

For gene expression analysis, total RNA was extracted from RAW264.7 cells or approximately 100 mg of lung tissue using the TRIzol-based method (Invitrogen) according to the standard protocol. The concentration and purity of the isolated RNA were verified before proceeding to cDNA synthesis. Quantitative real-time PCR (qPCR) was conducted on a LightCycler 480 platform (Roche) to determine the transcript levels of target genes. The relative mRNA abundance was quantified using the 2^−ΔΔCT^ method, with normalization to internal control genes. All specific primer sequences utilized in this study are provided in Supplementary Table S1.

### 2.12. Immunofluorescence Analysis

For lung sections, tissues were preserved in 4% paraformaldehyde and subsequently cryoprotected using a graded sucrose series. The tissues were then embedded in OCT compound (DZ2000, Leagene Biotechnology, Beijing, China) and cut into 5-μm-thick sections using a cryostat. To facilitate staining, these cryosections were incubated in a blocking solution containing 5% BSA and 0.5% Triton-X for 1 h at room temperature (RT). Primary antibody labeling was performed overnight at 4 °C using antibodies against CitH3 (ab5103, Abcam), F4/80 (MCA497G, Bio-Rad, Hercules, CA, USA), or Ly6G (31469, Cell Signaling Technology (CST), Danvers, MA, USA). Following the primary incubation, the sections were treated with appropriate fluorophore-conjugated secondary antibodies, including Alexa Fluor 488-labeled anti-rabbit (ab150077, Abcam) and Alexa Fluor 594-labeled anti-mouse (ab150080, Abcam), for 1 h. Nuclei were visualized by counterstaining with DAPI for 30 min at room temperature. All images were acquired using a confocal microscope (Olympus, Tokyo, Japan).

For in vitro imaging, RAW264.7 cells (with or without LPS stimulation) were rinsed with PBS and fixed in 4% paraformaldehyde for 20 min. Membrane permeabilization was achieved using 0.25% Triton X-100 for 15 min, followed by a 30 min blocking step with 1% bovine serum albumin at ambient temperature. The cells were then probed with an anti-CitH3 primary antibody at 4 °C overnight. After several PBS washes, the samples were labeled with an Alexa Fluor 488-conjugated secondary antibody (ab150077, Abcam) for 1 h. DAPI was applied to counterstain the nuclei. Images were captured and analyzed using Imaris 9.0 software (Bitplane, Belfast, UK), where MET formation was quantified based on the spatial colocalization of extracellular DNA (DAPI) and CitH3 fluorescence signals.

For the detection of METs, cells were sequentially stained with 500 nM SYTOX Green (S7020, ThermoFisher Scientific) at RT for 30 min to label extracellular DNA and then with DAPI. METs formation was quantified by determining the co-localized area of SYTOX Green and DAPI signal using Imaris 9.0.

### 2.13. Flow Cytometry Analysis

To prepare single-cell suspensions, lung tissues were subjected to enzymatic digestion at 37 °C for 30 min in RPMI-1640 medium supplemented with DNase I and collagenase D (or type IV). The dissociated cells were filtered through 70 µm cell strainers, followed by the elimination of erythrocytes using red blood cell lysis buffer. For immunophenotyping, cells were first incubated with surface antibodies against CD45 (BV605, 157217, BioLegend, San Diego, CA, USA) and F4/80 (FITC, 123107, BioLegend) for 30 min at 4 °C. After two consecutive PBS washes, the cells were processed with a commercial fixation and permeabilization kit. This enabled the subsequent intracellular labeling of iNOS2 (BV421, 404-5920-82, ThermoFisher), HIF-1α (APC-CY7, 17-7528-82, ThermoFisher), IL-1β (APC, 17-7114-80, ThermoFisher), or TNF-α (APC-CY7, 506343, BioLegend). All flow cytometric data were collected on a BD LSRFortessa™ system (BD Biosciences, Franklin Lakes, NJ, USA) and processed using FlowJo 10 software.

### 2.14. RNA Sequencing and Analysis

Total RNA was extracted from RAW264.7 cells using TRNzol Universal Reagent (TIANGEN Biotech, Beijing, China) according to the manufacturer’s instructions. For this purpose, RNA sequencing was performed by Hangzhou Kaitai Biotechnology (Hangzhou, China). Subsequently, bioinformatic analysis involved differential expression analysis using DESeq2 (FDR < 0.05), with results presented as a heatmap (OmicStudio, Shanghai, China). GO and KEGG enrichment analyses of DEGs were conducted using the R package clusterProfiler (version 4.2.0), with significance defined as *p* < 0.05.

### 2.15. Extracellular Acidification Rate (ECAR) Analysis

The ECAR of RAW264.7 cells was assessed using a Seahorse XF24 analyzer in conjunction with the Glycolysis Stress Test kit (Agilent Technologies, Santa Clara, CA, USA). Briefly, 1 × 10^5^ RAW264.7 cells were plated overnight. Prior to the assay, the culture medium was replaced with glucose-free, unbuffered XF assay medium (pH 7.4), and the cells were incubated in a non-CO_2_ incubator at 37 °C for 1 h for equilibration. Following the recording of baseline measurements, three compounds-glucose (10 mM), oligomycin (1 μM), and 2-DG (50 mM)-were sequentially introduced into the system at specific intervals. The resulting metabolic data were analyzed using the Wave Desktop software (version 2.6, Agilent Technologies).

### 2.16. Statistical Analysis

Data processing and statistical evaluations were performed with GraphPad Prism 9 software (GraphPad Software, San Diego, CA, USA). To determine differences between two experimental groups, Student’s *t*-tests were employed. For studies involving multiple groups or two independent variables, one-way or two-way ANOVA was utilized, respectively, followed by Tukey’s post hoc analysis for multiple comparisons. All quantitative results are expressed as mean ± SEM. Each experiment was conducted with a minimum of three independent biological replicates (litters), as detailed in the individual figure legends. Statistical significance was defined as *p* < 0.05.

## 3. Results

### 3.1. Extracellular Trap Formation Plays an Important Role in Acute Lung Injury in a Model of Neonatal Sepsis

**Figure 1:**
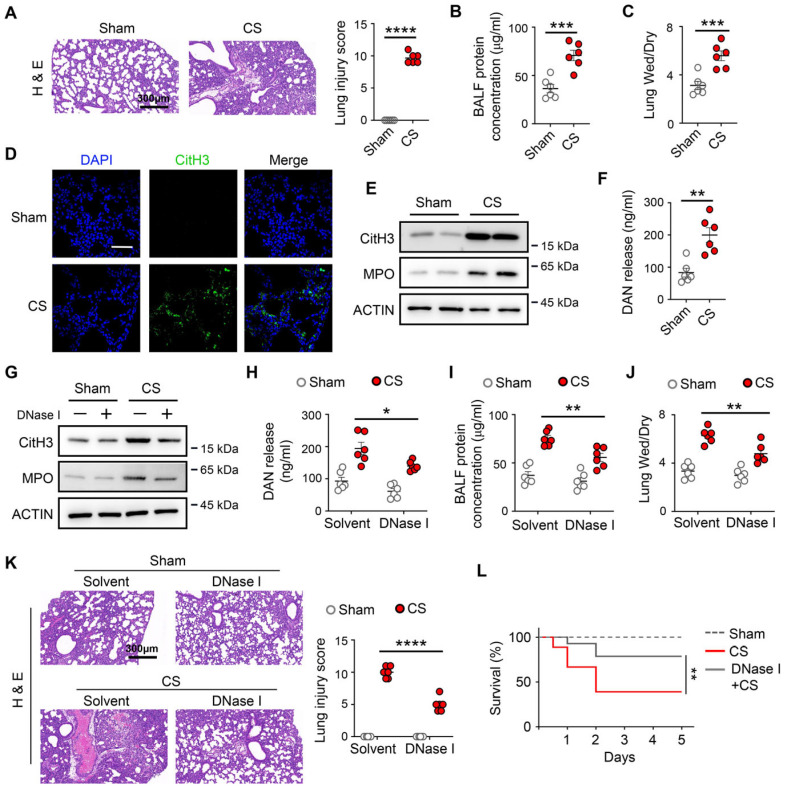
Extracellular traps are formed in the lung and contribute to injury in a neonatal sepsis model. Neonatal mice (postnatal day 5–7) were subjected to sepsis by intraperitoneal injection of cecal slurry (CS) or sham operation, and analyzed 24 h later. (**A**) Representative H&E staining of lung sections (scale bar: 300 μm) and lung injury scores (*n* = 6). (**B**) The protein concentration of BALF was measured (*n* = 3). (**C**) The wet/dry ratio of the lung was measured (*n* = 6). (**D**) Representative confocal microscopy images of lung sections. The sections show staining for DNA (blue) and CitH3 (green). Scale bars: 50 µm. (**E**) The protein levels of CitH3 and MPO in lung tissue were assessed by Western blot (*n* = 6). (**F**) Extracellular DNA levels in BALF as a marker for ETs were quantified (*n* = 6). (**G**–**K**) WT mice were pretreated with DNase I (5 mg/kg, i.p.), and then followed by sham or CS for 24 h. (**G**) The protein levels of CitH3 and MPO were measured in the lung by Western blot (*n* = 6). (**H**) Extracellular DNA levels in BALF were quantified (*n* = 6). The protein concentration of BALF (**I**) and the wet/dry ratio of lung (**J**) were measured (*n* = 6). (**K**) Representative H&E staining of lung sections (scale bar: 300 μm) and lung injury scores (*n* = 6). (**L**) The survival of sham and CS-treated neonatal mice with DNase I (5 mg/kg, i.p.) treatment (*n* = 10–18). Kaplan–Meier survival analysis with the log-rank test was used to compare survival rates in septic mice. * *p* < 0.05, ** *p* < 0.01, *** *p* < 0.001, **** *p* < 0.0001.

To determine whether ETs contribute to the pathogenesis of neonatal sepsis-induced acute lung injury (ALI) in newborn mice, neonatal mice subjected to cecal slurry-induced sepsis developed pronounced lung injury, as evidenced by histopathological changes, increased bronchoalveolar lavage fluid (BALF) protein levels, and elevated lung wet-to-dry weight ratios (Figure 1A–C). Consistent with the development of lung injury, markers of extracellular traps, including citrullinated histone H3 (CitH3) and myeloperoxidase (MPO), were markedly increased in lung tissue (Figure 1D,E), accompanied by elevated extracellular DNA levels in BALF (Figure 1F). Next, we assessed the effect of ET inhibitors on neonatal sepsis-induced ALI. We found that DNase I treatment, which degrades the scaffold structure of ETs, significantly decreased ET formation (Figure 1G) and BALF DNA release (Figure 1H) in the lung after the neonatal sepsis model. As expected, mice treated with DNase I suppressed BALF protein levels, lung wet-to-dry weight ratios, and attenuated lung injury, as well as improved the survival rates of neonatal septic mice (Figure 1I–L). Taken together, these results indicate that ETs contribute to neonatal sepsis-induced ALI.

### 3.2. The Formation of an Extracellular Trap from Macrophage Exacerbates ALI After Neonatal Sepsis

**Figure 2:**
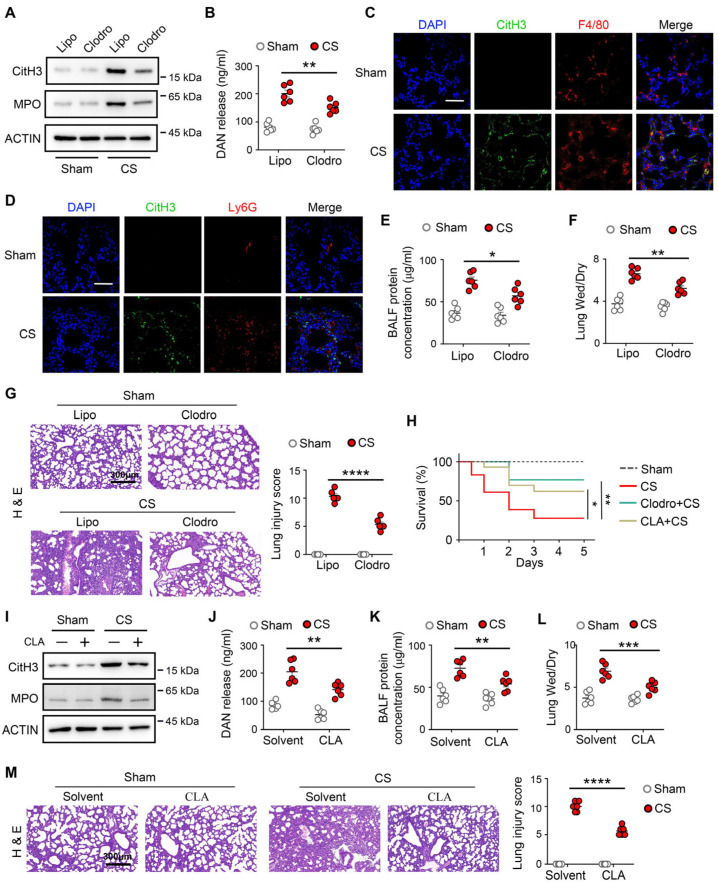
Macrophage extracellular trap formation is a key mediator of neonatal sepsis-induced acute lung injury. (**A**–**F**) Neonatal mice (postnatal day 5–7) were treated with PBS liposomal (Lipo) or clodronate liposomal (Clod, intraperitoneal injection), followed by sepsis for 24 h (*n* = 6). (**A**) The protein levels of CitH3 and MPO in lung tissue were measured by Western blot (*n* = 6). (**B**) Extracellular DNA levels in BALF were quantified (*n* = 6). (**C**) Representative confocal microscopy images of lung sections. The sections show staining for DNA (blue), CitH3 (green), and F4/80 (red). Scale bars: 50 µm. (**D**) Representative confocal microscopy images of lung sections. The sections show staining for DNA (blue), CitH3 (green), and Ly6G (red). Scale bars: 50 µm. The protein concentration of BALF (**E**) and the wet/dry ratio of lung (**F**) were measured (*n* = 6). (**G**) Representative H&E staining of lung sections (scale bar: 300 μm) and lung injury scores (*n* = 6). (**H**) The survival of sham and CS-treated neonatal mice with clodronate liposomal or Cl-amidine (CLA, 10 mg/kg, s.c. for 3 days) treatment (*n* = 10–18). (**I**–**M**) WT mice were pretreated with CLA (10 mg/kg, s.c. for 3 days) and then subjected to sham or CS challenge for 24 h. (**I**) The protein levels of CitH3 and MPO in lung tissue were measured by Western blot (*n* = 6). (**J**) Extracellular DNA levels in BALF were quantified (*n* = 6). The protein concentration of BALF (**K**) and the wet/dry ratio of lung (**L**) were measured (*n* = 6). (**M**) Representative H&E staining of lung sections (scale bar: 300 μm) and lung injury scores (*n* = 6). Kaplan–Meier survival analysis with the log-rank test was used to compare survival rates in septic mice. * *p* < 0.05, ** *p* < 0.01, *** *p* < 0.001, **** *p* < 0.0001.

Recent studies have shown that ETs are released from leukocytes, including neutrophils and macrophages [8,22], which are referred to as neutrophil extracellular trap (NETs) and macrophage extracellular trap (METs), respectively. To elucidate the origin of ETs release in neonatal sepsis-induced ALI, we used the anti-neutrophil antigen Ly6G and clodronate liposomes to deplete neutrophils and macrophages, respectively. Compared with PBS liposomal (Lipo), clodronate liposomal (Clodro) has been shown to deplete 80% macrophages in the lung (Figure S1A). Similarly, compared with anti-IgG, anti-Ly6G has been shown to deplete 80% neutrophils in the lung (Figure S1B). We found that macrophage depletion significantly decreased ET formation in the lung (Figure 2A), which was accompanied by lower DNA levels in the BALF (Figure 2B). In contrast, neutrophil depletion had no effect (Figure S1C,D). CitH3 colocalized with F4/80 (a macrophage marker) in the lung after neonatal sepsis (Figure 2C), whereas minimal co-localization between CitH3 and Ly6G (a neutrophil marker) was observed (Figure 2D), indicating that ET formation is primarily derived from macrophages rather than neutrophils in neonatal septic mice.

Furthermore, macrophage depletion attenuated lung injury and improved the survival rates in neonatal sepsis mice (Figure 2E–H). Also, treatment with METs inhibitor (Cl-amidine, a specific PADI4 inhibitor) had substantially decreased METs formation (Figure 2I) and BALF DNA release (Figure 2J) in the lung, which is accompanied by a marked improvement in both lung injury and survival rate in neonatal septic mice (Figure 2H,K–M). These findings indicate that METs represent a major contributor to extracellular trap formation and ALI in neonatal sepsis.

### 3.3. METs Promote Neonatal Sepsis-Induced ALI by Macrophage Activation

**Figure 3:**
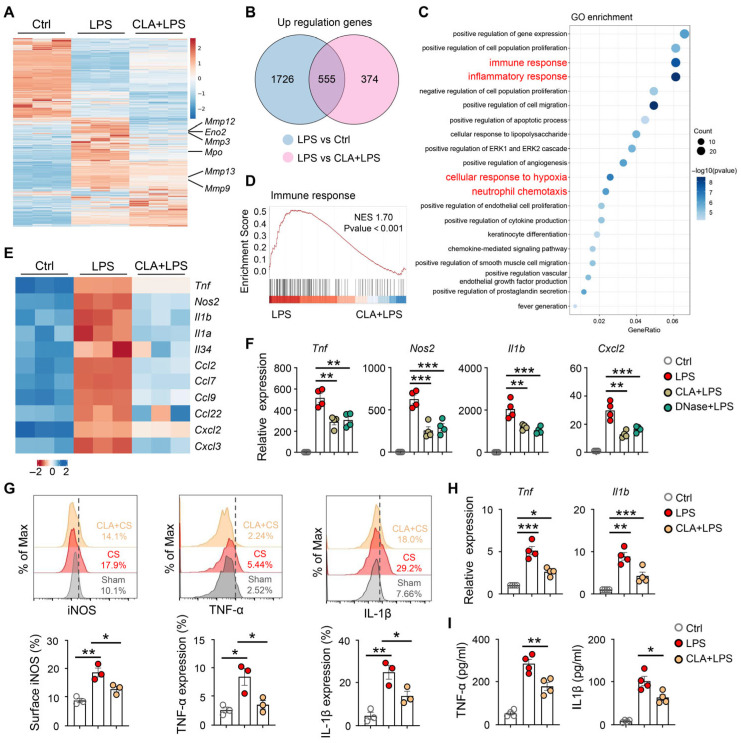
METs contribute to lung injury after the neonatal sepsis model associated with macrophage activation. (**A**–**E**) RNA-seq analysis of cultured RAW264.7 under control conditions, LPS-treated, or CLA + LPS-treated. (**A**) Heatmap of differentially expressed genes (DEGs). (**B**) Venn diagram of DEGs from LPS-treated versus CLA + LPS-treated. (**C**) GO enrichment analysis of the final 555 DEGs. (**D**) GSEA of the immune response gene set. (**E**) Heatmap showing differential expression of inflammatory-associated genes (*Tnf*, *Il1b*, *Il1a*), chemotaxis-associated genes (*Ccl2*, *Ccl7*, *Cxcl2*, *Cxcl3*), and macrophage polarization-associated gene (*Nos2*). (**F**) RAW264.7 cells were pretreated with CLA (200 μM) or DNase I (10 U/mL) for 1 h, followed by LPS for 12 h. The mRNA levels of *Tnf*, *Il1b*, *Cxcl2*, and *Nos2* were measured by qPCR (*n* = 4). (**G**–**I**) WT mice were pretreated with Cl-amidine (CLA, 10 mg/kg, s.c.) and then followed by sham or CS for 24 h. (**G**) The expression of TNFα, Il1β, and NOS2 was assayed in lung macrophages by FACS (*n* = 3). (**H**) The mRNA levels of *Tnf* and *Il1b* were tested in lung tissue (*n* = 4). (**I**) The concentration of TNF-α and Il1β in lung tissue was measured by ELISA. * *p* < 0.05, ** *p* < 0.01, *** *p* < 0.001.

To explore the function of METs in macrophages, we first confirmed METs formation after exposure to lipopolysaccharide (LPS) or phorbol 12-myristate 13-acetate (PMA, a stimulus for ETs formation) in macrophages in vitro (Figure S2A–E). Next, we performed RNA sequencing on macrophages under control conditions, LPS stimulation, or CLA pretreatment followed by LPS stimulation. As expected, LPS increased the expression of METs genes (including *Mmp12*, *Mmp3*, *Mmp13*, *Mmp9*, and *Mpo*) in macrophages in vitro, which could be decreased by CLA treatment (Figure 3A). The RNA sequencing analysis revealed 555 differentially expressed genes (DEGs) that were upregulated in LPS-treated macrophages (Figure 3B), which were enriched for pathways related to immune response, inflammatory response, and neutrophil chemotaxis by GO analyses (Figure 3C). Similarly, GSEA of RNA-seq data revealed a significant reduction in immune response, inflammatory response, and neutrophil chemotaxis genes in CLA-pretreated macrophages after exposure to LPS (Figure 3D and Figure S3A,B). Furthermore, CLA treatment led to diminished expression of a number of macrophage activation-associated genes after exposure to LPS, such as inflammatory-associated genes (*Tnf*, *Il1b*, *Il1a*), chemotaxis-associated genes (*Ccl2*, *Ccl7*, *Cxcl2*, *Cxcl3*), and macrophage polarization-associated gene (*Nos2*) (Figure 3E). These transcriptional changes are consistent with an activated macrophage phenotype, suggesting that METs formation amplifies macrophage activation.

To examine whether LPS-induced macrophage activation was dependent on METs, we pretreated macrophages with CLA or DNase I before LPS stimulation. We found that both CLA and DNase I treatment decreased the expression of *Tnf*, *Nos2*, *Il1b*, and *Cxcl2* mRNA after stimulating macrophages with LPS (Figure 3F), suggesting that blockage of METs production inhibits LPS-induced macrophage activation. To determine whether sepsis-induced ALI is caused by MET-mediated macrophage activation in vivo, we treated mice with CLA following sepsis. We found that treatment with CLA reduced TNFα, Il1β, and NOS2 expression in lung macrophages after sepsis (Figure 3G), which was accompanied by reduced inflammatory response in lung tissue (Figure 3H,I). Taken together, these findings suggest that METs promote macrophage activation, which induces lung injury after neonatal sepsis.

### 3.4. HIF-1α Is Associated with Glycolysis-Dependent METs Formation

**Figure 4:**
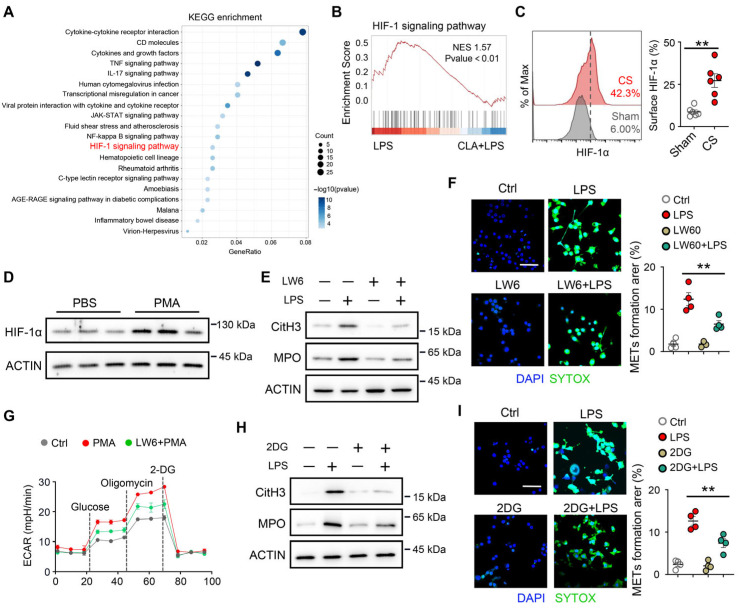
METs formation was dependent on HIF-1α–driven glycolysis. (**A**) KEGG enrichment analysis of the final 555 DEGs (as in Figure 3B). (**B**) GSEA of the HIF signaling pathway gene set. (**C**) HIF-1α levels in lung macrophages following sepsis (*n* = 6). (**D**) RAW264.7 cells were exposed to PMA (100 nM) for 12 h. The protein levels of HIF-1α were measured by Western blot (*n* = 4). (**E**,**F**) RAW264.7 cells were pretreated with 20 μM LW6 for 1 h, followed by LPS (100 ng/mL) for 12 h. (**E**) The protein levels of CitH3 and MPO were measured by Western blot (*n* = 4). (**F**) SYTOX Green staining was performed to observe the rate of METs and to analyze the MET rate (*n* = 4). (**G**) RAW264.7 cells were pretreated with 20 μM LW6 for 1 h, followed by PMA (100 nM) for 12 h. Extracellular acidification rate (ECAR) of RAW264.7 cells was measured (*n* = 3). (**H**,**I**) RAW264.7 cells were pretreated with 50 mM 2DG for 1 h, followed by LPS (100 ng/mL) for 12 h. (**H**) The protein levels of CitH3 and MPO were measured by Western blot (*n* = 4). (**I**) SYTOX Green staining was performed to observe the rate of METs and to analyze the MET rate (*n* = 4). ** *p* < 0.01.

Next, we investigate the mechanism underlying the formation of METs in macrophages. The 555 DEGs were enriched in pathways related to cellular response to hypoxia (Figure 3C) and HIF-1 signaling pathway (Figure 4A) according to GO and KEGG analyses. Furthermore, GSEA on the RNA-seq data revealed that the significant enrichment of the HIF-1 signaling pathway gene set (Figure 4B) or cellular response to hypoxia gene set (Figure S4A) following CLA-pretreatment. Notably, HIF-1α expression was upregulated in lung macrophages with CS stimulation (Figure 4C). We therefore examined whether METs formation was dependent on HIF-1α. We treated macrophages with PMA (a direct pharmacological inducer of extracellular trap formation) in vitro and found that HIF-1α expression was increased (Figure 4D). Next, we found that LW6, an inhibitor of HIF-1α, significantly reduced LPS-induced METs formation in macrophages (Figure 4E,F, and Figure S4B).

HIF-1α is a key driver of glycolytic metabolism, which is essential for macrophage activation [23,24,25]. Given the central role of glycolysis in macrophage activation, we examined whether HIF-1α–driven metabolic reprogramming regulates METs formation. We found that glycolytic genes (*Eno2*, *Hk1*, *Pfkfb3*, and *Slc2a1*) were upregulated in macrophages following LPS stimulation, which could be inhibited by CLA treatment (Figure S4C). Next, we investigated the role of glycolysis in METs formation and found that PMA stimulation led to elevated macrophage glycolytic capacity, which was abrogated following LW6 treatment (Figure 4G). Importantly, pharmacological inhibition of glycolysis by 2-deoxyglucose (2-DG) suppressed METs formation after LPS stimulation (Figure 4H,I, and Figure S4D). These results suggest a role for HIF-1α in glycolysis-dependent METs formation.

### 3.5. HIF-1α Is Involved in ENO2-Associated METs Formation

**Figure 5:**
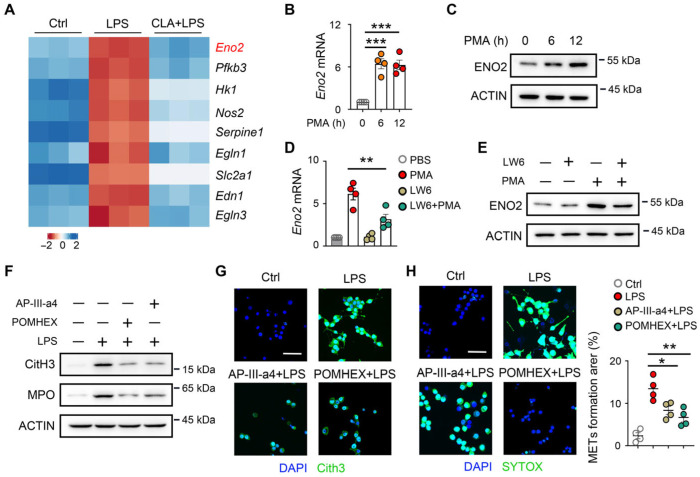
ENO2 contributed to HIF-1α–mediated METs formation. (**A**) The heatmap of the HIF-1 signaling pathway-associated gene. (**B**,**C**) RAW264.7 cells were exposed to PMA (100 nM) at different times. (**B**) The mRNA level of *Eno2* was measured by qPCR (*n* = 4). (**C**) The protein level of ENO2 was measured by Western blot (*n* = 4). (**D**,**E**) RAW264.7 cells were pretreated with LW6 (20 μM) for 1 h, followed by LPS (100 ng/mL) for 12 h. (**D**) The mRNA level of *Eno2* was measured by qPCR (*n* = 4). (**E**) The protein level of ENO2 was measured by Western blot (*n* = 4). (**F**–**H**) RAW264.7 cells were pretreated with AP-III-a4 (10 μM) or POMHEX (10 μM) for 1 h, followed by LPS for 12 h. (**F**) The protein levels of CitH3 and MPO were measured by Western blot (*n* = 4). (**G**) Immunofluorescence staining of CitH3 in RAW264.7 cells. Scale bar, 50 μm. (**H**) SYTOX Green staining was performed to observe the rate of METs and to analyze the MET rate (*n* = 4). * *p* < 0.05, ** *p* < 0.01, *** *p* < 0.001.

To further investigate the mechanism underlying HIF-1α-mediated METs formation in macrophages. The heatmap of HIF-1 signaling pathway-related (Figure 5A) and glycolysis process-related genes (Figure S4C) indicated that ENO2 was upregulated in LPS-treated macrophages compared with control and CLA + LPS-treated macrophages. Moreover, the mRNA and protein levels of ENO2 were elevated after PMA stimulation (Figure 5B,C), indicating that ENO2 might be associated with the HIF-1α–glycolysis mediated METs formation. Next, we found that LW6 inhibited the mRNA and protein levels of ENO2 after PMA stimulation (Figure 5D,E). To confirm the role of ENO2 in METs formation, macrophages were treated in vitro with AP-III-a4, a candidate ENO2-targeting compound, or POMHEX, a selective ENO2 inhibitor [26,27]. We found that AP-III-a4 or POMHEX treatment significantly reduced the formation of METs in macrophages in vitro (Figure 5F–H). These findings suggest that ENO2 contributes to HIF-1α–mediated METs formation.

### 3.6. Inhibition of HIF-1α–ENO2 Signaling Reduces Lung METs Formation and Improves Lung Injury and Survival in Neonatal Sepsis Model

**Figure 6:**
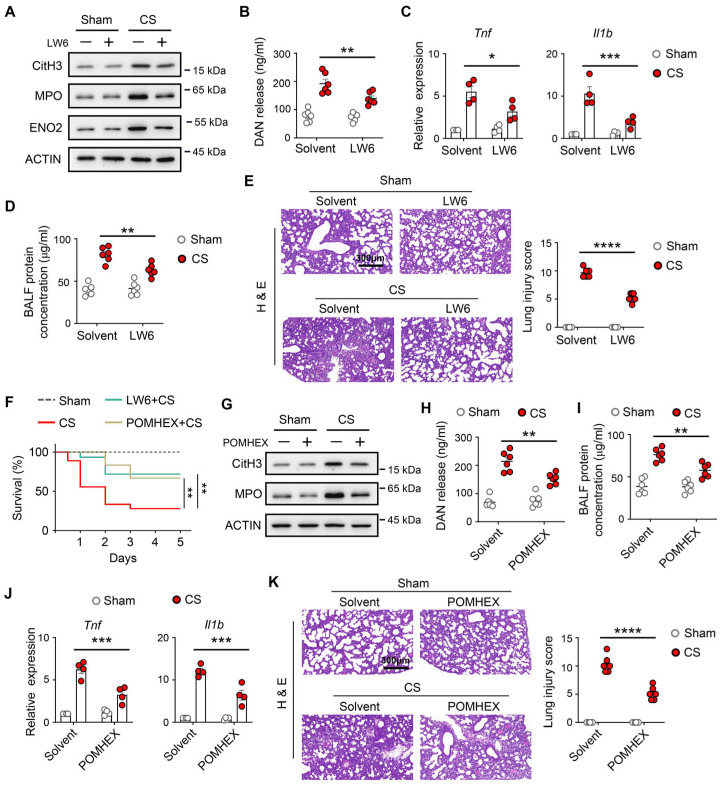
Inhibition of HIF-1α or ENO2 protected against neonatal sepsis-induced acute lung injury. (**A**–**J**) Neonatal mice (postnatal day 5–7) were pretreated with 20 mg/kg LW6 (**A**–**E**) or 20 mg/kg POMHEX (**G**–**K**), and then followed by sepsis for 24 h. (**A**,**G**) The protein levels of CitH3, MPO, and ENO2 were measured in the lung by Western blot (*n* = 6). (**B**,**H**) The extracellular DNA release was quantified in BALF (*n* = 6). (**C**,**J**) The mRNA levels of Tnf and Il1b were tested in lung tissue (*n* = 4). (**D**,**I**) The protein concentration of BALF was measured (*n* = 6). (**E**,**K**) Representative H&E staining of lung sections (scale bar: 300 μm) and lung injury scores (*n* = 6). (**F**) The survival of sham and CS-treated neonatal mice with LW6 or POMHEX treatment (*n* = 10–18). The survival rates of septic mice were analyzed using the Kaplan–Meier method with the log-rank test. * *p* < 0.05, ** *p* < 0.01, *** *p* < 0.001, **** *p* < 0.0001.

Our findings suggested that HIF-1α–ENO2 signaling in lung macrophages might be a valuable therapeutic approach for the neonatal sepsis model. We examined the therapeutic potential of targeting the HIF-1α–ENO2 signaling with a drug in animal models of neonatal sepsis. We found that treatment LW6 reduced METs formation (Figure 6A), BALF DNA release (Figure 6B), and ENO2 expression (Figure 6A) in lung after neonatal sepsis model, which was accompanied with reduced inflammatory response in lung tissue (Figure 6C), and attenuated the lung injury (Figure 6D,E), as well as improved the survival rates in neonatal septic mice (Figure 6F). Also, treatment with POMHEX had the same effect (Figure 6F–K). Taken together, these data indicate that METs formation, which could be induced by HIF-1α–ENO2 signaling, causes macrophage activation, thereby contributing to lung injury.

## 4. Discussion

Acute lung injury (ALI) is a frequent and severe complication of this condition, yet specific therapeutic strategies remain limited [1,2]. The pathophysiology of neonatal sepsis-induced ALI involves distinct aspects of the developing immune system. Newborns, particularly preterm infants, exhibit altered immune and metabolic responses to infection, which likely influence the course of the disease [28,29,30]. This study investigates the formation of macrophage extracellular traps (METs) as a potential driver of lung injury in this specific context. We describe a mechanistic pathway in which the HIF-1α–ENO2 axis promotes glycolysis, a metabolic shift associated with innate immune cell activation [31], and promotes METs formation. Furthermore, our data suggest that METs may further amplify macrophage activation, potentially creating a sustained inflammatory response.

The release of extracellular traps (ETs) is an immune effector mechanism that can contribute to tissue damage in various pathologies. Beyond their role in infection, METs have been identified as mediators in sterile inflammatory conditions. For example, in rhabdomyolysis-induced acute kidney injury, METs released from platelet-activated macrophages directly contribute to tubular epithelial cell death and renal dysfunction [10]. In cancer, necroptosis-driven METs formation in pancreatic cancer has been shown to facilitate tumor metastasis by altering the tumor microenvironment [9]. These studies indicate that METs can act as mediators of tissue damage through mechanisms such as direct cytotoxicity and inflammation. Our findings contribute to this understanding by highlighting their role in the developing lung. In the early phase of neonatal sepsis-induced lung injury, METs, rather than neutrophil extracellular traps (NETs), appeared to be the predominant form of ETs, as evidenced by the specific reduction in injury following macrophage depletion. Importantly, our data demonstrate that while neutrophils are recruited to the neonatal lung following sepsis, they do not exhibit significant ET formation at this early phase. This suggests that although neutrophils are present and may exert other immunological functions, the early phase of ET-mediated acute lung injury in this model is primarily driven by macrophage-derived METs. This cellular specificity may be influenced by the unique neonatal pulmonary immune landscape, which is characterized by a predominance of inflammatory M1-like alveolar macrophages and a relatively immature neutrophil effector function, including reduced NET-forming capacity [32,33,34]. Whether this initial METs subsequently influences neutrophil recruitment, or whether these recruited neutrophils subsequently initiate NET formation at later stages of disease progression, represents an important question for future research, similar to cellular interactions observed in other inflammatory settings, such as severe asthma [35].

A key finding is the role of the HIF-1α–ENO2–glycolysis axis as a metabolic regulator of METs in neonatal sepsis. Our data indicate that sepsis-induced HIF-1α stabilization is associated with increased expression of the glycolytic enzyme ENO2, contributing to a metabolic state that supports METs release. This is consistent with the link between macrophage function and cellular metabolism [31]. Notably, a similar HIF-1α–dependent glycolytic shift in alveolar macrophages, driven by Wnt/β-catenin signaling during viral infection, has been shown to promote inflammation [36]. Our study suggests that in neonatal sepsis, HIF-1α promotes ENO2-mediated glycolysis, which regulates METs formation. The observed reduction in METs formation following inhibition of HIF-1α (with LW6) or ENO2 (with POMHEX) supports this axis as a potential therapeutic target.

Our data further suggest that METs may contribute to a self-reinforcing cycle of inflammation by enhancing macrophage activation. Inhibition of MET formation led to a reduction in the expression of classic M1-associated genes and key chemokines. This implies that components of METs (e.g., histones, DNA) may act as damage-associated molecular patterns (DAMPs), helping to sustain inflammatory signaling. This potential vicious cycle, where initial signals trigger METs, and METs in turn promote further macrophage activation, could exacerbate the progression of ALI in neonates. This concept is consistent with studies showing that extracellular histones are cytotoxic and contribute to organ injury in sepsis [6,37]. From a therapeutic perspective, our findings highlight the potential of targeting the METs pathway in early neonatal sepsis. Current adjuvant therapies, such as intravenous immunoglobulins, have shown limited or uncertain efficacy in improving mortality rates [38]. Therefore, strategies aimed at inhibiting MET formation, or its upstream regulators, could represent a novel approach. Such strategies might be complementary to existing ones that protect endothelial function [39] or inhibit other forms of cell death, such as necroptosis [12].

This study has several limitations. First, although RAW264.7 cells were used for mechanistic investigations, they are derived from adult macrophages and may not fully reflect the biological characteristics of neonatal macrophages. The neonatal immune system exhibits distinct functional and metabolic features, which may influence macrophage responses during sepsis. Future studies using primary neonatal macrophages will be important to validate these findings. Second, while we utilized specific pharmacological inhibitors to identify the HIF-1α–ENO2 axis, these findings require further validation using cell-specific genetic models, such as siRNA- or CRISPR-mediated targeting of Hif1a or Eno2. Future studies employing genetic approaches will be important to further confirm the role of this pathway. The relative contributions of different macrophage subsets to MET formation need elucidation. The precise DAMP receptors through which METs feedback to activate macrophages remain to be defined. Emerging research identifies specific serum metabolite signatures in neonatal sepsis that correlate with the disease state and treatment response [40]. Looking forward, the integration of mechanistic insights with clinical biomarkers holds promise.

## 5. Conclusions

This study identifies macrophage extracellular traps (METs) as an important early contributor to lung injury in a neonatal sepsis model. We suggest that METs formation is associated with a HIF-1α-ENO2-associated glycolytic program and is linked to enhanced macrophage inflammatory activation, thereby amplifying pulmonary injury. These findings provide mechanistic insight into the role of METs in the pathogenesis of neonatal sepsis-associated acute lung injury. Targeting components of this pathway may represent a potential avenue for therapeutic intervention.

## Data Availability

All data supporting the findings are provided within the article or are available from the corresponding authors upon reasonable request.

## Supplementary Materials

The following supporting information can be downloaded at: https://www.mdpi.com/article/10.3390/biomedicines14051145/s1, Figure S1: Neutrophil depletion did not affect the formation of ETs; Figure S2: LPS or PMA induced METs formation in vitro; Figure S3: METs formation was associated with macrophage activation; Figure S4: HIF-1α-driven glycolysis contributed to METs formation; Table S1: List of primers used qRT-PCR.
