# Supplementary material for: HIF-1α Promotes Macrophage Extracellular Trap Formation and Exacerbates Acute Lung Injury in Neonatal Sepsis
Source: Biomedicines. 2026 May 18;14(5):1145. doi: 10.3390/biomedicines14051145 (PMC13204621; doi:10.3390/biomedicines14051145)
Supplement: Supplementary file 1 [file biomedicines-14-01145-s001.zip › biomedicines-4253685-supplementary.pdf]

HIF-1α Promotes Macrophage Extracellular Trap Formation and Exacerbates Acute Lung Injury in Neonatal Sepsis

Huiling Zhang [1, †], Wei Huang [2, †], Xinlong Dai [3, †], Jundi Zheng [4], Xinyao Jiang [3], Yutao Yang [3], Hanhui Zhong [3, *], Guang Yang[1, *]

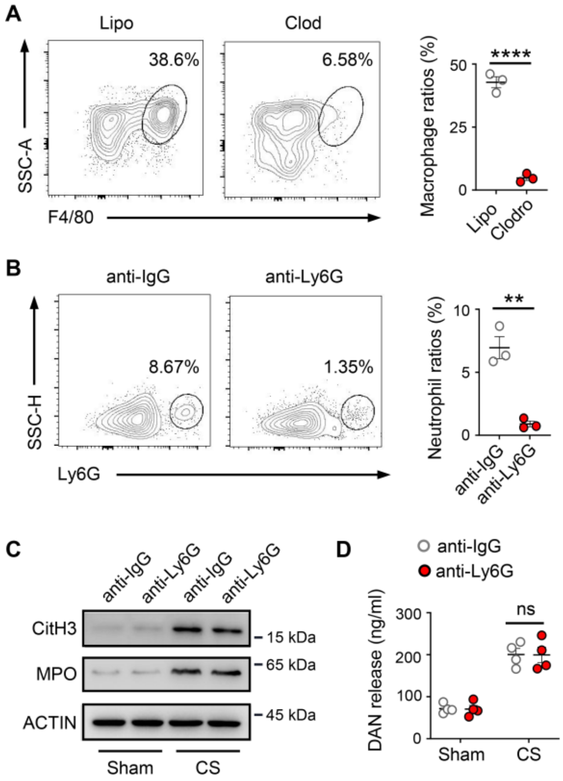

Supplementary Figure S1. Neutrophil depletion did not affect the formation of ETs. (A) Neonatal mice (postnatal day 5-7) were treated with PBS liposomal (Lipo) or clodronate liposomal (Clod, intraperitoneal injection). Flow cytometry analysis of lung macrophage ratio in live, CD45+ cells (n=3). **(B-D)** Neonatal mice (postnatal day 5-7) were injected with 50 μg mouse IgG Isotype control (IgG) or anti-Ly6G antibody (i. p.), followed sham and sepsis for 24 h. (B) Flow cytometry analysis of lung neutrophile ratio in live, CD45+ cells (n=3). (C) The protein levels of CitH3 and MPO in lung tissue were measured by western blot (n=4). (D) Extracellular DNA levels in BALF were quantified (n=4). **P<0.01, ****P<0.0001, ns: not significant.

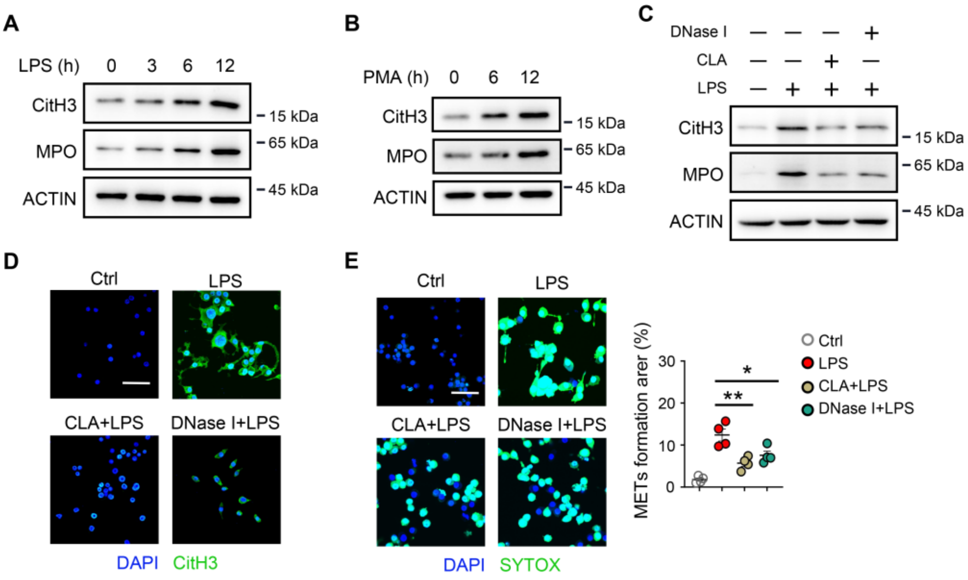

Supplementary Figure S2. LPS or PMA induced METs formation in vitro. (A, B) RAW264.7 cells were exposed to LPS (100 ng/mL) or PMA (100 nM) at different time. The protein levels of CitH3 and MPO were measured by western blot (n=4). **(C-E)** RAW264.7 cells were pretreated with CLA (200 μM) or DNase I (10 U/mL) for 1 h, followed by LPS (100 ng/mL) for 12 h. (C) The protein levels of CitH3 and MPO were measured by western blot (n=4). (D) Immunofluorescence staining of CitH3 in RAW264.7 cells. Scale bar, 50 μm. (E) SYTOX Green staining was performed to observe the rate of METs and analysis of METs rate (n=4). *P<0.05, **P<0.01.

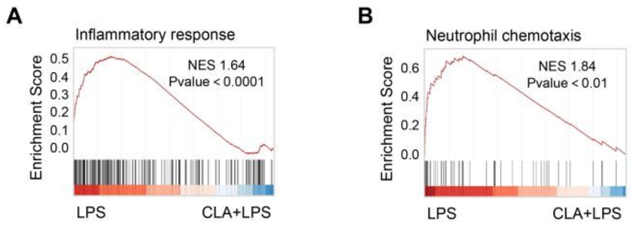

Supplementary Figure S3. METs formation was associated with macrophage activation. RNA-seq analysis of cultured RAW264.7 with control, LPS-treated, or CLA+LPS-treated. GSEA of inflammation response gene **(A)** and neutrophil chemotaxis gene **(B)** set.

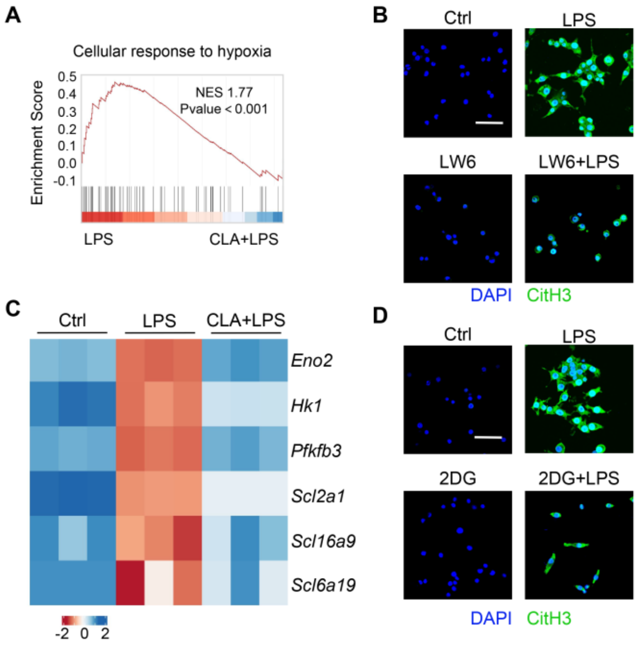

Supplementary Figure S4. HIF-1α-driven glycolysis contributed to METs formation. (A) GSEA of Cellular response to hypoxia gene set. **(B)** RAW264.7 cells were pretreated with 20 μM LW6 for 1 h, followed by 100 ng/mL LPS for 12 h. Immunofluorescence staining of CitH3 in RAW264.7 cells. Scale bar, 50 μm. **(C)** Heatmap showing differentially expression of glycolysis-associated genes *(Eno2, Hk1, Pfkfb3, Slc2a1, Slc16a9, Slc6a19*). **(D)** RAW264.7 cells were pretreated with 50 mM 2DG for 1 h, followed by 100 ng/mL LPS for 12 h. Immunofluorescence staining of CitH3 in RAW264.7 cells. Scale bar, 50 μm. Data shown represents four independent experiments.

Supplementary Table S1 | List of primers used qRT-PCR.

Primers		Species
18S-Forward	GTAACCCGTTGAACCCCATT	Mouse
18S-Reverse	CCATCCAATCGGTAGTAGCG	
Nos2-Forward	GAGACAGGGAAGTCTGAAGCAC	Mouse
Nos2-Reverse	CCAGCAGTAG TTGCTCCTCTTC	
Tnf-Forward	AAGCCTGTAGCC CACGTCGTA	Mouse
Tnf-Reverse	GGCACCACTAGTTGGTTGTCTTTG	
Il1b-Forward	GAAATGCCACCTTTTGACAGTG	Mouse
Il1b-Reverse	TGGATGCTCTCATCAGGACAG	
Eno2-Forward	CGTTACTTAGGCAAAGGTGTCC	Mouse
Eno2-Reverse	CTCCAGCATCAGGTTGTCCAGT	
Cxcl2-Forward	CCTGGTTCAGAAAATCATCCA	Mouse
Cxcl2-Reverse	CTTCCGTTGAGGGACAGC	
